# Psychometric evaluation of the Disabilities of the Arm, Shoulder and Hand (DASH) with Dupuytren’s contracture: validity evidence using Rasch modeling

**DOI:** 10.1186/1471-2474-15-361

**Published:** 2014-10-30

**Authors:** Nancy J Forget, Christina Jerosch-Herold, Lee Shepstone, Johanne Higgins

**Affiliations:** École de santé publique de l’Université de Montréal (ESPUM), Université de Montréal, Montreal, Canada; School of physical and occupational therapy, McGill University, Montreal, Canada; Centre de la main, Centre hospitalier de l’Université de Montréal (CHUM), Montreal, Canada; School of Health Sciences, Faculty of Medicine and Health Sciences, University of East Anglia, Norwich, UK; Norwich Medical School, Faculty of Medicine and Health Sciences, University of East Anglia, Norwich, UK; École de réadaptation, Université de Montréal, Montreal, Canada; Centre de recherche interdisciplinaire en réadaptation du Montréal métropolitain (CRIR), Montreal, Canada; Institut de réadaptation Gingras-Lindsay de Montréal, Montreal, Canada

**Keywords:** Outcome assessment, Activities of daily living, Disability evaluation, Psychometric properties, Validity evidence, Item-response theory, Rasch analysis, Dupuytren’s contracture, Musculoskeletal disorder

## Abstract

**Background:**

Dupuytren’s contracture is a progressive, fibroproliferative disorder that causes fixed finger contractures and can lead to disability. With the advances of new therapeutic interventions, the necessity to assess the functional repercussions of this condition using valid, reliable and sensitive outcome measures is of growing interest. The Disabilities of the Arm, Shoulder and Hand (DASH) is one frequently used patient-reported outcome measure but its reliability and validity have never been demonstrated specifically for a population affected with Dupuytren’s contracture. The objective of this study was to evaluate the psychometric properties of the DASH, with focus on validity evidence using the Rasch measurement model.

**Methods:**

Secondary analysis was performed on data collected as part of a randomised clinical trial. One hundred fifty-three participants diagnosed with Dupuytren’s contracture completed the DASH at four time points (pre-op, 3, 6 and 12 months post-op). Baseline data were analysed using traditional analysis and to test whether they adhered to the expectations of the Rasch model. Post-intervention data were subsequently included and analyzed to determine the effect of the intervention on the items.

**Results:**

DASH scores demonstrated large ceiling effects at all time points. Initial fit to the Rasch model revealed that the DASH did not adhere to the expectations of the Rasch partial credit model (χ^2^ = 119.92; *p* < 0.05). Multiple items displayed inadequate response categories and two items displayed differential item functioning by gender. Items were transformed and one item deleted leading to an adequate fit. Remaining items fit the Rasch model but still do not target well the population under study.

**Conclusions:**

The original version of the 30-item DASH did not display adequate validity evidence for use in a population with Dupuytren’s contracture. Further development is required to improve the DASH for this population.

**Electronic supplementary material:**

The online version of this article (doi:10.1186/1471-2474-15-361) contains supplementary material, which is available to authorized users.

## Background

Dupuytren’s disease is a chronic and progressive musculoskeletal condition affecting the hands [1] and is characterized by a progressive thickening of the palmar fascia. It results in the creation of nodules and cords at the level of the palm and/or fingers, which can lead to a gradual flexion contracture and permanent finger extension restriction. Its point prevalence in a male Caucasian population is estimated to be 8.8% for working age (French male civil servants; approx. mean age 45 y.o) [2] and tends to increase with age, estimated to be 39% in Icelandic males aged 70-74 (29% with nodules only; 10% with finger contracture) [3]. To correct the deformities occasioned by Dupuytren’s contracture (DC), a surgical approach is most commonly used so far [1]. Recently, there has been a growing scientific interest in less invasive interventions for DC, such as percutaneous needle fasciotomy [4] or collagenase injection [5]. In order to determine treatment effectiveness, the assessment of functional performance is now recognized as an important outcome measure to include in clinical trials related to upper extremity conditions. One of the most widely used patient-reported outcome measuring this construct is the *Disabilities of the Arm, Shoulder and Hand* (DASH) [6]. The DASH has been extensively studied, its reliability and validity demonstrated in many different populations, has been translated in multiple languages and its relation to the *International Classification of Functioning, Disability and Health* (ICF) has been verified [7, 8]. It has been shown to be psychometrically robust, free of charge and United States population norms are available [7, 9]. The construct validity of the DASH was demonstrated in samples regrouping various upper extremity conditions, and no floor or ceiling effects (ie: referring to a large distribution near the bottom and top scores respectively) were observed in a sample of people with either wrist\hand or shoulder problems [10]. Based on traditional analyses, the results of multiple studies support the use of the DASH as an appropriate measure of functional performance in persons with proximal humeral fractures [11], shoulder disabilities [12, 13], ulnar neuropathy at the elbow [14], rheumatoid arthritis [15], work-related musculoskeletal complaints [16], and thumb osteoarthritis [17]. A recent review reported numerous studies that used the DASH to assess functional performance with a population affected by DC [18]. However, to the best of our knowledge, no validation study has ever been undertaken specifically with this population.

Current thinking defines validity less as a property of the test but rather as an appraisal of the meaning of the test score based on empirical evidence, dependent not only on the test’s characteristics, but also on its respondents’ characteristics and on the evaluative context [19]. According to the *Standards for educational and psychological testing* [20], the validation process of a test should include 5 sources of empirical evidence in order to provide a comprehensive validity argument. Evidences based on test content, based on response processes, based on internal structure, based on relations to other variables and based on consequences of testing can be demonstrated with traditional arguments and with item response theory (IRT), including the Rasch modeling [21–23]. Rasch is a statistical model which describes the relationship between persons’ ability and individual item’s level of difficulty [24]. This model transforms patient-reported outcome measure’ ordinal scales into interval scales and performs a linear transformation of the raw scores depicting the latent trait being measured as a continuum [25]. The individual items’ locations along this continuum mark their level of difficulty and the persons’ locations represent their ability level on the latent trait and fit to the model provides the *empirical evidence to support how well the items* measure that latent trait [26]. Multiple studies have analysed the DASH based on samples including various musculoskeletal conditions using Rasch modeling: to generate a clinically useful collection form [27], to compare with other measures [28], to develop a shorter version [29], to examine its factor structure [30, 31], and to estimate its psychometric properties [31]. To the best of our knowledge, only one study looked at the DASH’s psychometric properties in relation to a specific condition, the sample composed of people affected by multiple sclerosis [32].

The objective of this study was to evaluate the psychometric properties of the DASH, with focus on validity evidence using partial credit Rasch measurement model [33] in a sample of people diagnosed with DC.

## Methods

### Sample and data collection

Secondary analysis were performed on data collected as part of a multi-centre, pragmatic, randomised controlled trial on the clinical effectiveness of static night-time splinting after fasciectomy or dermofasciectomy (SCoRD trial - registered as an International Standard Randomised Controlled Trial ISRCTN57079614) [34, 35]. Patients were eligible if they were 18 years and older, developed DC in one or more fingers and were waiting for a fasciectomy or dermofasciectomy. Ethics approval of the original trial was obtained in July 2007 by the Cambridgeshire 2 Research Ethics Committee (REC 07/Q0108/120) and by the Research Governance and Ethics Committee of each participating hospital. All participants gave written informed consent [35].

Data on personal factors, such as age and gender, and DASH total scores from 4 time points (before the surgery (baseline) and at 3-, 6- and 12-months post-surgical intervention) were retrieved and analysed for 153 participants.

### Outcome measure: DASH

The *Disabilities of the Arm, Shoulder and Hand* (DASH) is a 30-item regional patient-reported outcome measure designed to measure functioning and symptoms (http://www.dash.iwh.on.ca/). The DASH scores on a 5-point Likert scale (1-*No difficulty* to 5-*Unable*) and total score ranges from 0 (no disability) to 100 (severe disability). It comprises of 21 items on functioning and 9 items on symptom severity.

### Statistical analyses

Descriptive statistics were used to portray our sample’s characteristics, scores’ distribution and items’ response profile. Cross-sectional reliability estimate were performed, with a Cronbach’s α between 0.7-0.95 considered good [36]. Analyses were completed using IBM SPSS Statistics for Windows, Version 20.0 (IBM Corp. 2011, Armonk, NY).

### Validity evidence using Rasch analysis

The American Educational Research Association proposed a different conceptualisation of validity that offers guidelines for *developing a scientifically sound validity argument to support the intended interpretation of test scores and their relevance to the proposed use* [20]. Viewed as a unitary concept, validity can be appreciated by demonstrating five distinct, but not mutually exclusive, types of evidence: evidence based on test content, on response pattern, on internal structure, on relations to other variables and on consequences of testing [20]. Using these definitions, Lim & al. [21] demonstrated the usefulness of using Rasch analysis in order to appraise the different types of validity evidence.

### Rasch analysis

Items of the DASH administered prior to surgery were analysed with RUMM2030 software [37] using the Rasch partial credit model which is suited for measures with scales composed of ≥2 ordered response categories (e.g. from *no difficulty* to *unable*) [38]. As Rasch analysis requires that a single construct is measured, unidimensionality of the DASH was assessed a priori by Principal Component Analysis (PCA), as recommended and performed elsewhere [39–41], using the Proc Factor procedure with SAS version 9.1 (SAS Institute Inc. Cary, NC, USA). First, data were evaluated to determine whether the pre-requisites for conducting PCA were met (normality, interval-level measurement, random sampling and bivariate normal distribution) [42]. Because the DASH is scored on an ordinal scale, PCA was carried out using a polychoric correlation as an input. Selection of the final number of factors was based on established rules: eigenvalues (ϵ) >1 [43], scree test [44] and percentage of the common variance explained by the different components.

### Evidence based on test content

Related to content validity, validity evidence based on test content is demonstrated through proper targeting of the items and the absence of gaps along the latent trait continuum [21]. Because Rasch analysis places items and persons along the same linear continuum, if no items are located in the vicinity of the persons’ level of ability or if there are important gaps between the difficulty levels of the items, the ability of these persons cannot be estimated with precision. This type of evidence was investigated by the use of different statistics (eg: person ability estimates, test information function) and by inspection of the item-person map for proper targeting of the items to the persons. If the items are clustered on the right and the persons on the left, or vice-versa, it is indicative of mistargeting. It can also be verified by comparing the mean location score obtained for the persons’ ability with that of the value of zero set as a default for the items’ difficulty. The presence of gaps in the items’ location along the continuum was also inspected.

### Evidence based on response processes

Related to construct validity, evidence based on response processes examines the adequacy of the type of responses in relation to the construct being measured [45]. This can be performed by looking at the extent to which the subjects’ responses correspond to the expectations of the Rasch model [46] and is done through the examination of several ‘fit indices’ or fit statistics. First, the *global model fit statistic,* reported as a chi-square, is used to assess the overall fit with a statistically significant (*p* <0.05) result indicating an ill-fitting model [47]. *Item* and *person standardized fit residuals* should be located between ±2.5 logits to be considered as fitting the model [48]. They are expected to have a mean of zero and a standard deviation of 1. Their corresponding chi-squares and F-statistics must be non-significant (*p* >0.05). We further investigated this type of evidence by looking at the adequacy of response categories. For items scored on an ordinal scale (>2 categories), responses should be adequately distributed across the item’s response categories, and this can be indicated as a minimum of 10 responses per response category [49]. Also, a well functioning scale should have all of its composing response categories demonstrating the highest probability of being endorsed at different level of difficulty, as examined with the probability curves. The thresholds are those points along a theoretical continuum of item difficulty where the probability of a person responding either 0-or-1, and 1-or-2 respectively, is equally likely.

### Evidence based on internal structure

Also related to construct validity, this type of evidence examines the relationship between the items and the measured construct and within the measure’s items [21]. Evidence based on internal structure is demonstrated if acceptable *person* and *item fit statistics* are obtained and items are not displaying any *differential item functioning* (DIF). A true measure must be invariant and the probability of “success” on an item must not be affected by the person’s personal characteristics. Items displaying DIF demonstrate different probabilities depending on the group of persons being assessed (e.g. men vs. women) and violate the property of invariance inherent to the Rasch model. DIF can be detected using the *item characteristic curves* (ICC) that describe the relationship between an item’s difficulty (X axis) and a person’s ability (Y axis) where as a person’s ability increases, the probability of getting a “correct” answer also increases. If the location of the curves differs while the slopes are identical, it indicates the presence of a uniform DIF: the difference between groups is constant across all ability levels. Whereas, when the slopes are not parallel or cross each other, it is indicative of non-uniform DIF: the differences between groups vary across the ability continuum. The variables that were examined for the presence of DIF were the time of evaluation, age, gender, clinical characteristics (Woodruff grade [50]) and randomisation group.

### Evidence based on consequences of testing

This type of evidence examines the consequences of the measurement (of the testing procedure and of the interpretation of scores obtained) on the people on whom the test is administered [20]. This type of evidence can be estimated by looking at both the anticipated and unanticipated benefits of the measurement and the differential consequences amongst subgroups of the population [51]. Even if Rasch analysis does not directly address this type of evidence, the results obtained by the DIF and targeting are useful to draw a judgment on this type of evidence [21]. As an example, if the test demonstrates DIF by gender, this could imply that it might be more difficult for men (or women) to get a higher (or lower) score on the test, which could lead to discrimination based on gender.

### Reliability evidence

Reliability of the DASH was also examined using Rasch. In RUMM2030, the reliability index, also called the *person separation index*, is interpreted as a Cronbach’s α and indicates how well the items can discriminate persons in different levels of ability [25] with an estimate >0.8 deemed satisfactory [52].

### Change over time

Post-intervention evaluations (3-, 6-, and 12-months post-intervention) were subsequently racked by appending the post intervention item responses onto the baseline item responses [53, 54]. In other words, each participant’s scores is entered four times: once for every testing occasion. This procedure generates item difficulty estimates for baseline and post intervention assessments and determines whether the intervention had an impact on item locations. For the sole purpose of performing this analysis, a proper fitting model needs to be obtained. Misfitting items are deleted starting with the most misfitting, based on their fit statistics or the presence of DIF.

## Results

### Sample characteristics

Baseline data from 153 participants were analysed. Mean age was 67 (SD 10) and composed of 119 men (78%) and 34 women. All participants underwent fasciectomy (n = 138) or dermofasciectomy (n = 15) and the vast majority had multiple digits and/or multiple joints affected (Woodruff Grade 2- *MCP only*: n = 10; Grade 3- *MCP and PIP, single digit*: n = 87; Grade 4- *as Grade 3 in multiple digits*: n = 56).

### Score distribution and traditional reliability estimate

Baseline: Mean DASH score was 15.9 (SD 14.5) with a positive skew for our score distribution. Five percent of participants obtained a total score of 0 (meaning *no disability*). Two items demonstrate a substantial amount of missing values with one item reaching an unacceptable threshold, defined as >15% [55] (5% of missing values for *item* 19- *Recreational activities in which you move your arm freely*; 15.7% for *item* 21- *Sexual activities*). The number of responses per response option is also positively skewed, with proportions ranging from 32% (item “open jar”) to 83,7% (items “turn a key” and “sexual activities”) of respondents that utilized the *no difficulty* (or equivalent) category. All items demonstrated underutilized scale categories (<10 responses per category), ranging from 1 to 3 of the 5 available response categories, and all of which are situated to the hardest extreme of the scale (from *moderate difficulty* to *unable*).

Other time points: The mean DASH scores were 10.3 (SD 12.9), 7.6 (SD 11.1), 6.7 (SD 12.3) at 3-, 6- and 12-months respectively, with proportions of total scores of 0 (*no disability*) of 17%, 28% and 35% of our sample at 3-, 6- and 12-months post-intervention respectively.

Traditional reliability analysis revealed a Cronbach’s α of 0.95 which represents an excellent estimate of internal consistency.

### Principal component analysis

The random sampling and normality assumptions were met as each subject only contributed one score on each variable and skewness and kurtosis coefficients did not exceed ±2.0 for the majority of variables (>60%) [56]. Although other assumptions were inconsistently met due to the ordinal nature of the data, the results of the PCA were suitable to identify the number of dimensions measured by the DASH and the items potentially unrelated to a one-dimensional concept. The visual examination of the scree plot indicates the presence of one strong factor. Furthermore, based on the eigenvalues, one predominant factor similarly stands out, but some items are also loading on 3 other factors (Table 1). The first factor explains 60% while the second, third and fourth factors explain 6%, 5% and 4% of the variance respectively. As items predominantly load on the first factor, none of the items were removed following this first *triage* and all were included for the Rasch analysis.

**Table 1:** **Factor loadings from principal component analysis of the 30 items**

	Items	Factor loadings*			
		Factor 1	Factor 2	Factor 3	Factor 4
1.	Open a tight or new jar	0.80	-0.36		
2.	Write	0.72	-0.38	0.32	
3.	Turn a key	0.83	-0.42		
4.	Prepare a meal	0.83			
5.	Push open a heavy door	0.77			
6.	Place an object on a shelf above head	0.78		-0.30	
7.	Do heavy household chores	0.93			
8.	Garden or do yard work	0.83			
9.	Make a bed	0.86			
10.	Carry a shopping bag or briefcase	0.86			
11.	Carry a heavy object (over 10 lbs)	0.80			
12.	Change a lightbulb overhead	0.83			
13.	Wash or blow dry hair	0.78			
14.	Wash your back	0.79	0.32		
15.	Put on a pullover sweater	0.90			
16.	Use a knife to cut food	0.75	-0.40		
17.	Recreational activities which require little effort	0.75			
18.	Recreational activities with force or impact	0.81			
19.	Recreational activities which move arm freely	0.82			
20.	Manage transportation needs	0.79			
21.	Sexual activities	0.63			
22.	Interference with normal social activities	0.56	0.35		-0.62
23.	Limitation in work or other regular daily activities	0.57			
24.	Pain in arm, shoulder or hand	0.66		0.60	
25.	Pain when performing specific activity	0.78		0.52	
26.	Tingling	0.56			0.52
27.	Weakness	0.79			
28.	Stiffness	0.61	0.45		
29.	Difficulty sleeping	0.73			
30.	Feeling less capable, less confident or less useful	0.67			
	Eigenvalue	17.91	1.67	1.46	1.16

*Only loading of ≥0.3 are included.

### Rasch analyses

To facilitate the interpretation of results, recoding of the scale was performed in RUMM2030 by reversing the scores associated with scale’s difficulty level - categories now ranging from 0- *Unable* to 4- *No difficulty*.

**Figure 1:**
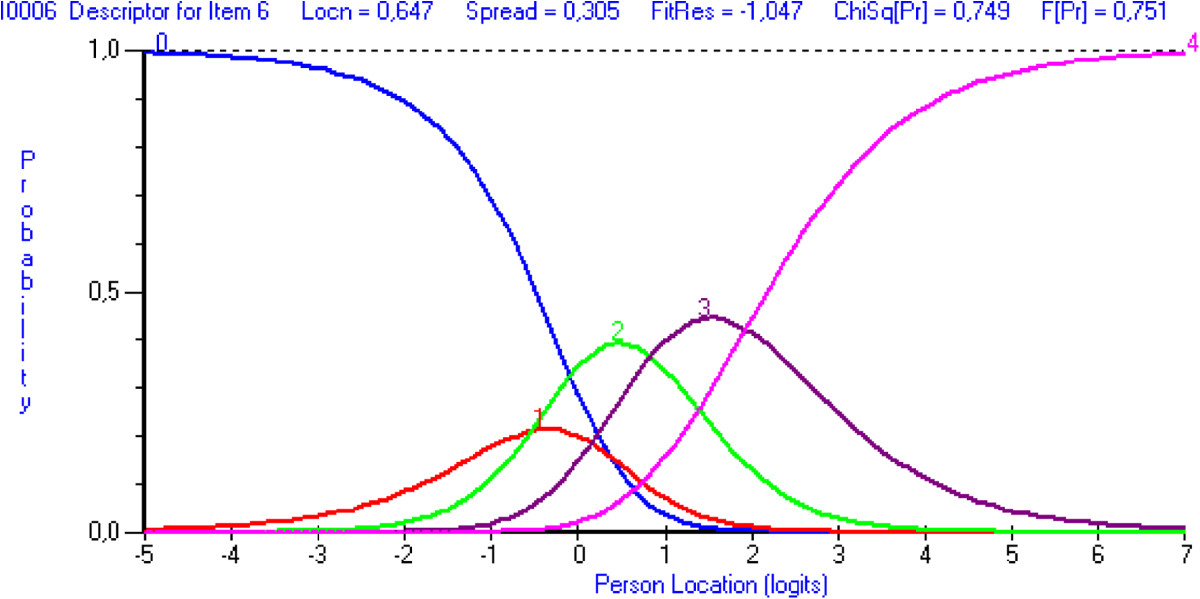
**Category probability curve displaying disordered 5-point response options for item 6-** ***Place on object on a shelf above your head.*** *Note that the response categories were recoded from 0 – *Unable* to 4 – *No difficulty*. Figure demonstrates that response category 4- *Severe difficulty* (corresponding to curve 1) is clearly underused.

**Figure 2:**
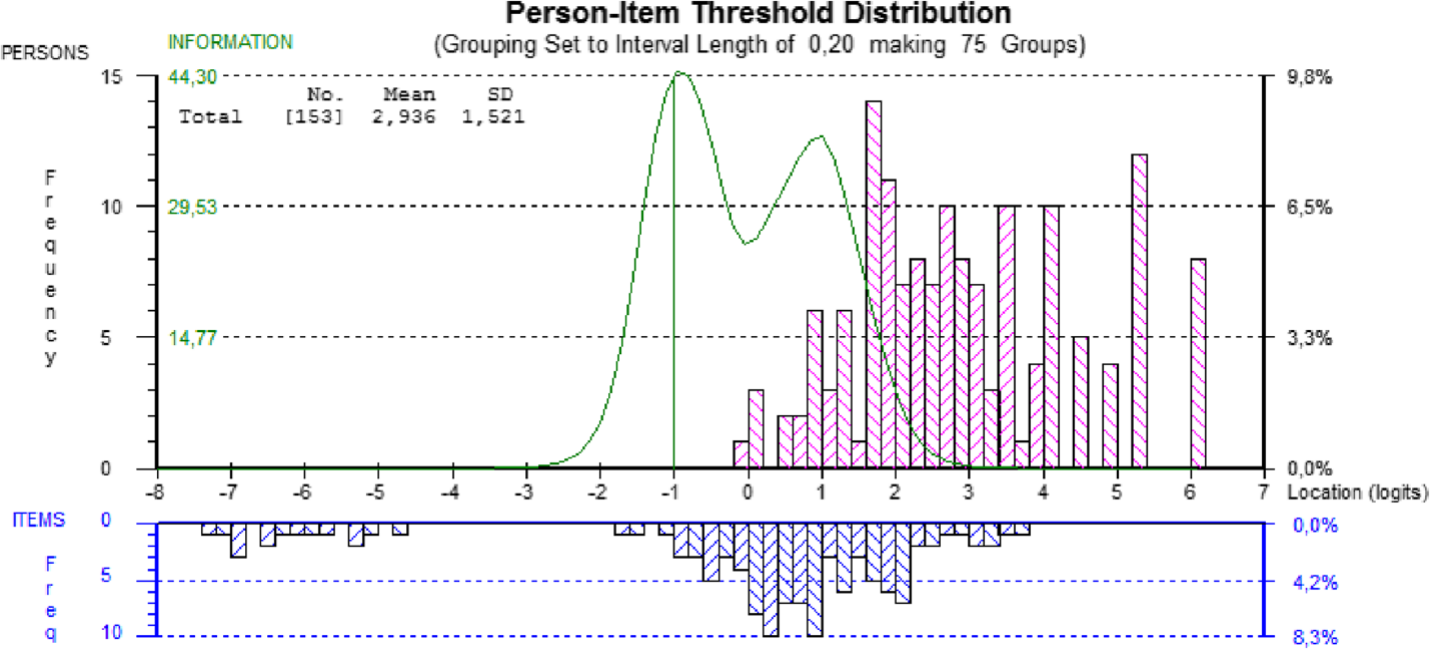
**Item-person threshold distribution map of the DASH; baseline evaluation.** The horizontal axis, scaled in logits, denote ability from least ability at the left to most ability at the right and the vertical axis denotes the proportion of subjects or items. The bars represent the distribution of subjects and items at each location. The curve represents the test *information function*, its highest peak representing the ability level at which persons are measured with the least amount of error.

When looking for the evidence based on response processes, the initial fit of the baseline data produced a significant item-trait interaction (χ^2^: 119.92; probability: *p* <0.05), indicating that the DASH does not meet the expectations of the Rasch model. Two items (28 and 30) misfit the model’s expectations based on fit residual values above 2.5, and five more items (7, 8, 9, 21 and 26) also had a significant chi-square statistics or F-statistics (Table 2). Eleven persons had residuals outside the recommended range (above or below 2.5). When looking at the adequacy of response categories, items 6, 12, 13, 17, 19-21 and 30 demonstrated disordered thresholds, with scoring categories that are clearly underused (see Figure 1 for example).When looking at the evidence based on test content and consequences of testing, the DASH’s level of difficulty of its composing items does not adequately target the level of ability of the persons. This is demonstrated by the item-person map that shows some gaps along the continuum, mainly located between -2 and -4 logits and above 4 logits (Figure 2). The mean person location is 2.936 while it should be located near the mean location of the items that is set at 0 by default.

**Table 2:** **The 30 items and their Rasch measurement properties by location order**

					Chi-square		F-stat	
	Items	Location	SE	Fit Residual	Value	***p***-value	Value	***p***-value
3.	Turn a key	-1.37	0.18	-1.149	3.953	0.139	2.833	0.062
20.	Manage transportation needs	-1.279	0.166	-0.475	1.932	0.381	0.864	0.424
15.	Put on a pullover sweater	-1.27	0.144	-1.57	4.864	0.088	2.902	0.058
4.	Prepare a meal	-1.219	0.154	-1.863	3.585	0.167	2.335	0.101
9.	Make a bed*	-1.13	0.143	-2.106	7.508	0.023	6.288	0.002
2.	Write	-1.051	0.162	0.177	1.805	0.406	0.658	0.519
29.	Difficulty sleeping	-1.017	0.137	0.365	0.067	0.967	0.066	0.936
10.	Carry a shopping bag or briefcase	-0.857	0.127	-1.072	2.319	0.314	1.049	0.353
26.	Tingling*	-0.817	0.134	2.186	8.396	0.015	2.299	0.104
22.	Interference with normal social activities	-0.808	0.139	1.213	4.652	0.098	1.024	0.362
8.	Garden or do yard work*	-0.701	0.13	-2.199	6.767	0.034	4.432	0.014
23.	Limitation in work or other regular daily activities	-0.657	0.13	-0.221	3.192	0.203	1.406	0.249
27.	Weakness	-0.495	0.124	-0.073	3.467	0.177	1.319	0.271
24.	Pain in arm, shoulder or hand	-0.434	0.127	1.662	2.928	0.231	1.191	0.307
25.	Pain when performing specific activity	-0.423	0.121	-0.097	0.791	0.673	0.417	0.660
5.	Push open a heavy door	0.197	0.135	-1.156	3.168	0.205	1.933	0.148
16.	Use a knife to cut food	0.252	0.142	-0.176	0.794	0.672	0.22	0.803
13.	Wash or blow dry hair	0.375	0.126	-0.996	0.306	0.858	0.182	0.833
17.	Recreational activities which require little effort	0.376	0.141	-0.364	2.547	0.280	1.861	0.159
6.	Place an object on a shelf above head	0.647	0.127	-1.047	0.578	0.749	0.287	0.751
7.	Do heavy household chores*	0.813	0.119	-2.302	7.891	0.019	5.143	0.007
14.	Wash your back	0.923	0.112	-0.559	0.837	0.658	0.34	0.712
21.	Sexual activities*	0.923	0.127	1.937	16.732	0.000	2.796	0.065
12.	Change a lightbulb overhead	0.944	0.118	-0.384	3.054	0.217	1.355	0.261
11.	Carry a heavy object (over 10 lbs)	1.094	0.11	-0.171	0.697	0.706	0.475	0.623
19.	Recreational activities which move arm freely	1.132	0.111	-0.867	1.026	0.599	0.741	0.478
**28.**	Stiffness*	1.223	0.109	**3.315**	11.746	0.003	4.221	0.017
18.	Recreational activities with force or impact	1.277	0.111	-0.14	0.718	0.698	0.246	0.782
1.	Open a tight or new jar	1.639	0.111	0.37	0.582	0.747	0.165	0.848
**30.**	Feeling less capable, less confident or less useful*	1.714	0.098	**2.937**	13.022	0.001	2.839	0.062

Positive location (measure) numbers mean harder items; negative location means easier items.

Abbreviation: SE, standard error.

Bolded items: outside the ±2.5 range for fit residuals.

*fit residuals with significant chi-square and\or F-statistics (*p* <0.05).

When looking at evidence based on internal structure and based on consequences of testing, the presence of DIF was explored and was deemed to be present if analyses of variance were significant (Bonferroni-corrected; *p* <0.000617). Items 5- *Push open a heavy door* and 11- *Carry a heavy object (over 10 lb)* demonstrated uniform DIF by gender with women scoring more towards the *unable* response category of the scale on these two items despite having the same level of ability as men. The person separation index obtained is 0.90, indicating excellent internal consistency reliability.

**Figure 3:**
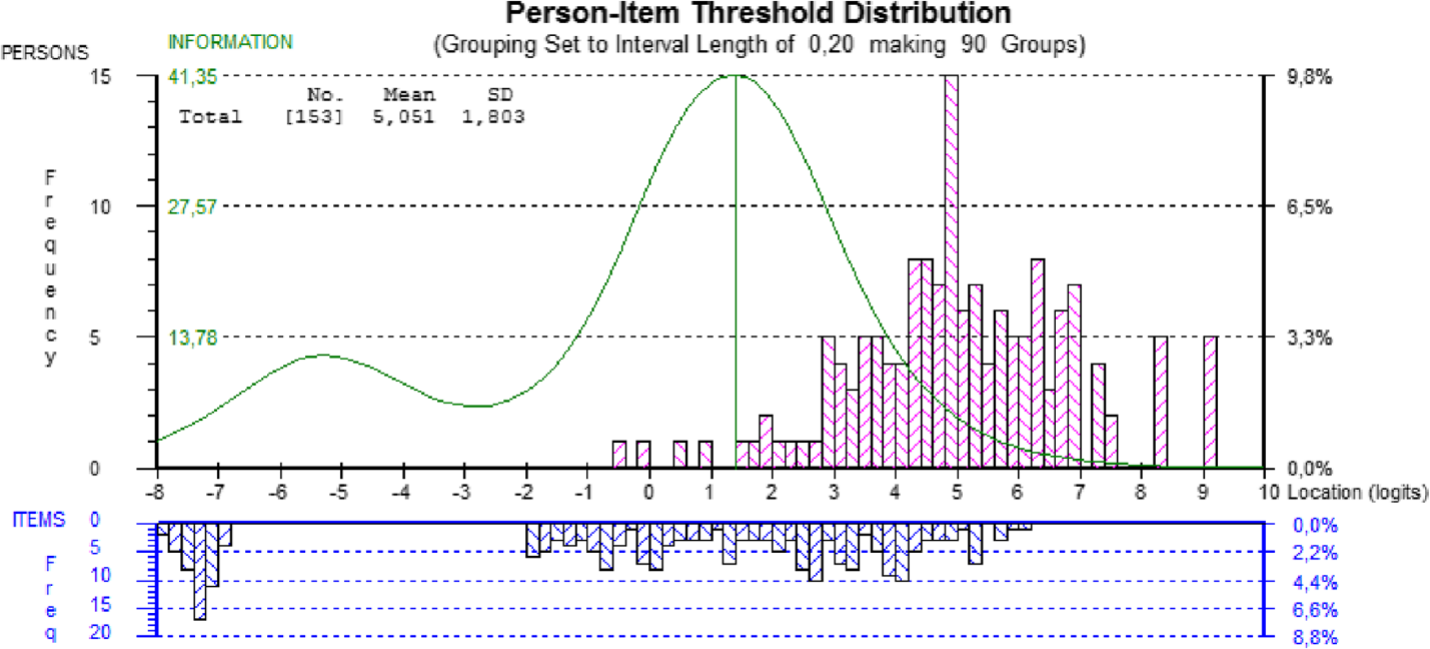
**Item-person threshold distribution map of the modified DASH; racked data.**

To examine the change in difficulty estimates over time, racking of data was performed and fit to the Rasch model was examined. Because the initial fit of the racked data produced a significant item-trait interaction with several misfitting items and persons, an attempt at transforming the DASH was made to meet the Rasch model expectations and thus allowing for interpretation of the results. Response categories were changed for all 30 items based on the criteria for optimizing category effectiveness [49]. All items were re-scored to have 3-response categories (categories 2-3 and 4-5 were combined respectively while category 1 remained), except for item 21 that was dichotomized for the 3-, 6- and 12-month evaluations and item 20 that was dichotomized for the 12-month evaluation. Once all items had ordered thresholds, all fit statistics, standardized residuals, chi-square and *F*-statistics, were re-examined. Item 26- *Tingling* still displayed misfit and was therefore deleted. The global fit statistic (χ^2^: 250.62; *p* = 0.191) confirmed that the 29 items of the modified DASH, scored with 2- or 3-response categories, work well together to measure upper extremity function in persons with DC. The unidimensionality of the modified DASH was supported by the principal component analysis of the residuals. Indeed, the first component explained less than 10% of the variance, confirming that all the information in the data is explained by the latent measure [57]. However, as depicted by the item-person map in Figure 3, the items are still mistargeting the persons with a mean person’s location of 5.051 (SD 1.803) in relation to the mean location of the items that is set at 0 by default.

**Figure 4:**
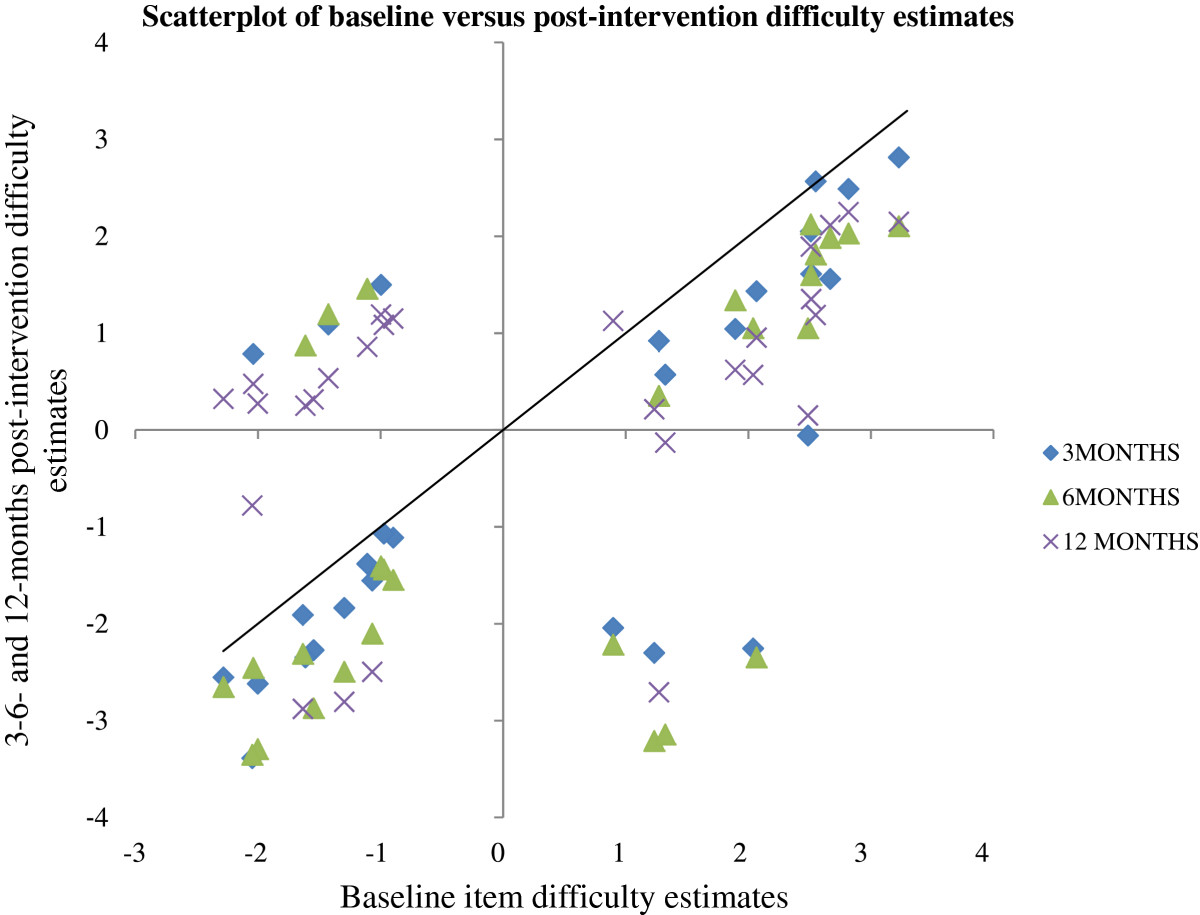
**Scatterplot of baseline versus post-intervention (3-, 6- and 12-month) items' difficulty estimates.** The black line indicates no change in item difficulty. Items that became easier after the surgical intervention are located below this line.

In order to visualize the change in item difficulty estimates, the baseline estimates were plotted against all other evaluation time points (Figure 4) [54]. The difficulty estimates that join on the ‘no change’ line, represented as the solid black line on the Figure, represent items that keep the same level of difficulty with time. Correspondingly, estimates that join below the line represent items that got easier and those joining above the line represent items that became harder. As persons undergoing surgery are expected to do better, the majority of items should become less difficult and be located below the ‘no change’ line, which is not the case here especially at 12 months post-surgery with 12 of the 29 items situated above the line.

## Discussion

To the best of our knowledge, this is the first study to look at the psychometric properties of the DASH specifically with a population affected by DC and we found that the original version of the 30-item DASH did not display adequate validity evidence for use with this population.

We first estimated the internal consistency of the DASH and found, with both analyses, excellent estimates that are consistent with those found in other studies [7, 58].

To evaluate the validity of the DASH, we used an original conceptualization that facilitates the analyses and interpretation of the results generated by the Rasch model. Our results do not support the different types of validity evidences, hence demonstrating that the DASH, in its present format, is not an ideal measure for this population.

Using a novel conceptualisation of validity [20], the results of our study offers a lot of information on the relevance of the DASH with a DC population. We first verified the factor structure of the DASH and found that the items were predominantly loading onto one factor, which allowed us to pursue Rasch analysis. However, similarly to other studies [30, 31], the items also loaded onto additional factors. As found by Kennedy et al. [7], the items that relate to more than one factor are mainly associated with symptoms, thus reviving the debate whether these items should be considered separately to those pertaining to functioning.

Based on the results of our traditional and Rasch analyses, evidence based on test content cannot be supported by our results. Our sample at baseline consisted of people waiting for surgery and thus certainly experiencing some functional difficulties. However, the mean DASH score obtained at baseline was 15.9, which is more consistent with the results obtained in the general US population (10.1 SD 14.7) than for people with diverse upper-extremity conditions (43.9 SD 22.9; n = 200) [7]. Moreover, the total scores’ distribution is positively skewed, indicative of a large ceiling effect. This affects the ability of the DASH to measure functioning and symptoms with precision and to discriminate higher functioning individuals. This effect could be partly explained by the ease of performance of some items for people affected by this pathology. Items like 6- *Place an object on a shelf above your head* or 14- *Wash your back* are likely to be easy to perform for these people because these tasks do not rely mainly on the biomechanics of the fingers for their realization. Moreover, the high rate of missing values for two items, but especially for item 21- *Sexual activities*, raises doubts about the relevance of including this item in the questionnaire.

Furthermore, an important gap in the items’ location along the continuum can be observed. This gap greatly hinders the ability of the DASH to assess with precision people that would be located around this level of ability. We also observed that the level of difficulty of the items of the DASH is not targeting well the ability level of our participants affected by DC. As pointed out by Hagquist et al. [59], targeting is crucial for a good measurement and mistargeting limits the ability of the measure to differentiate people along the latent trait. Again by observing the item-person map, we first see that a large proportion of persons are located considerably above the highest levels of item threshold, showing that a greater proportion of participants are too highly functioning to be picked up by the items. The person ability estimate has a mean of 2.936, which is far above the recommended 0 location, and is an indicator that this sample of participants are too able for the difficulty of the DASH’s items. Demonstrated through the Test Information Function (Figure 2), the DASH is most useful for people with an ability level around a logit of -1. We can also observe that the curve tapers off at around -3 and +3 units, points beyond which the persons’ ability cannot be measured with precision. Moreover, the bulk of items’ location is situated between -2 and +4 logits, whereas the persons’ location is from 0 to +6 logits. Finally, the presence of items at the far left side also demonstrates that some items are deemed too easy for a population affected by DC, which puts a burden on the patients to answer items that are irrelevant for them.

The results of our study do not support the evidence based on response processes. This was demonstrated by a significant item-trait interaction, an indication that the DASH does not meet the expectation of the Rasch model. Eleven persons and seven items were identified as not meeting the requirements of the model with residuals located beyond acceptable limits (±2.5) or by having significant chi-square or F-statistics. Interestingly, almost all of the misfitting items have actually been identified as problematic activities or impacting quality of life by patients affected by DC [60]. This phenomenon could partly be explained with a stereotypic view of gender task allocation that some of these activities can be regarded as carried out predominantly by women and therefore might be more or less relevant for males (ie: *house chores* and *making a bed*) which are the ones mainly affected by this disease. Also, some of the activities covered by these items might be too broad or the wording confusing, leaving room for the respondent’s own interpretation of the question and potentially distorting the response distribution. Perhaps fragmenting the activity (eg: instead of *Garden* revise as *Mow your lawn with a lawnmower)* or providing a definition (eg: defining what is meant by *stiffness* or *tingling*) could improve the respondents’ understanding and decrease individual interpretation of the items. We previously raised the issue that item 21- *Sexual activity* can be open to personal interpretation as to whether it could be regarded as referring to the physical or to the emotional aspect of sexuality (re: inability to perform physical acts or referring to sexual desire) which could have impacted the way the participants responded to this item [8].

A rating scale diagnosis was also performed. First, as recommended by Bond and Fox [25], the number of responses per category was considered based on the results of our traditional analysis. It clearly demonstrates that, for our sample of people affected by DC, the response categories *unable* and *severe difficulty* are highly underutilized with a very high proportion of respondents using the *no difficulty* response category, which is consistent with our hypothesis that the DASH’s items are too easy for this population. Based on our Rasch analysis, eight items had disordered response thresholds. For these items, this clearly demonstrates that some response categories never have the highest probability of being endorsed by the participants, which is also indicative that the scale contains too many response options.

Even if never done specifically with a DC population, the results of our Rasch analyses are consistent with results obtained from studies looking at other conditions. Targeting a population affected by multiple sclerosis, Cano and al. [32] found that the DASH showed misfit of thirteen items and disordered item response threshold for 9 items. They concluded that the DASH should be revised to improve its psychometric performance when used with a population affected by multiple sclerosis. Two other studies performed Rasch analysis with the DASH with larger samples composed of persons affected by diverse upper-extremity musculoskeletal or neurological disorders [30, 31]. Both found that three [30] and four [31] items showed poor fit to the Rasch model, with items 21- *Sexual Activities* and 26- *Tingling* being problematic in both studies, which is consistent with our results.

Again, the results of our study are not able to support evidence based on internal structure. As discussed previously, multiple items and persons demonstrated inadequate fit statistics, which is indicative that the persons and the items are not performing as expected by the Rasch model. Also, based on our knowledge, this is the first study to explore the DASH for DIF. Two items displayed DIF by gender. These items were found to both possess the qualifier “heavy” (items 5- *Push open a* heavy *door* and 11- *Carry a* heavy *object (over 10 lb)*).

The last evidence considered, evidence on consequences of testing, also cannot be supported. Again, the presence of DIF by gender demonstrates that the 2 items do not have the same meaning for men and women. Even if having similar levels of functioning, women tend to answer as being less able to lift or push heavy objects then men and is consistent with the fact that women tend to self-report lower self-perception of physical capacity than men [61]. Moreover, all of our analyses (eg: the presence of the large ceiling effect, mistargeting of the items, disordered response thresholds) suggest that the DASH would need revision to be used with a population affected by DC.

Numerous DC-related studies have used the DASH as primary outcome measure [35, 62–70]. However, the results of our study clearly demonstrate the limits of the DASH when used to measure disability and functioning with a population affected by DC. We modified the DASH in order to obtain a proper fit. After transformation, the 29 remaining items may serve as a basis for the development of a revised DASH specific for DC. However, this modified DASH is still too easy and therefore items with a higher level of difficulty and items that fill gaps should be created and further co-calibrated onto the same linear continuum. By a process of equating and anchoring, new items can be added and calibrated and improve the psychometric properties of the revised measure.

The results of the racked data when compared in time showed that the DASH’s items have a good correlation between baseline and post-intervention items difficulty estimates. This clearly demonstrates that the level of difficulty remains stable in time, with the easiest items at baseline remaining the easiest items after the intervention. However, a proportion of items became more difficult for participants, especially at 12 months post-intervention when compared with baseline. This could be indicative of the recurrence of the disease in some participants. However, recurrence was not measured in this trial because of the lack of consensus on what is a recurrence and how it is measured. Reported recurrence rates in the literature range from 5% up to 71% after a partial or total fasciectomy (at 24 to 120 months) [1].

The results of our study may have been impacted by our relatively small sample size. The presence of adequate targeting of the items to the sample, the sample size required to perform a Rasch analysis should have included at least 200 observations in order to yield stable person and item estimates (±0.5 logit at 95% confidence level) and based on an expected standard error level of ±0.1 [71]. However, our sample was large enough in order to appreciate the appropriateness of the DASH with a population affected by DC and was composed of persons of various stages of the disease.

## Conclusions

Even if extensively studied and internationally recognized and utilized, the DASH might not be the most appropriate measure when assessing function with people affected by DC. The DASH should be revised and further tested with people affected with DC in order to validate its use with this specific population or an alternative disease-specific measure needs to be developed and used in this population. Clinicians and researchers should be careful when deciding which outcome measure is the best to use for their population. Using an outcome measure that is not valid can have serious repercussions; it may invalidate the results of a study and pose an unnecessary burden on the patient. Even if studies like ours are not available for each outcome measure and in relation to every population, professionals should ascertain the validity of the chosen outcome prior to the intervention.

## Authors’ original submitted files for images

Below are the links to the authors’ original submitted files for images. Authors’ original file for figure 1 Authors’ original file for figure 2 Authors’ original file for figure 3 Authors’ original file for figure 4
