# Supplementary figures and images for: Psychometric evaluation of the Disabilities of the Arm, Shoulder and Hand (DASH) with Dupuytren’s contracture: validity evidence using Rasch modeling
Source: BMC Musculoskelet Disord. 2014 Oct 30;15:361. doi: 10.1186/1471-2474-15-361 (PMC4228176; doi:10.1186/1471-2474-15-361)

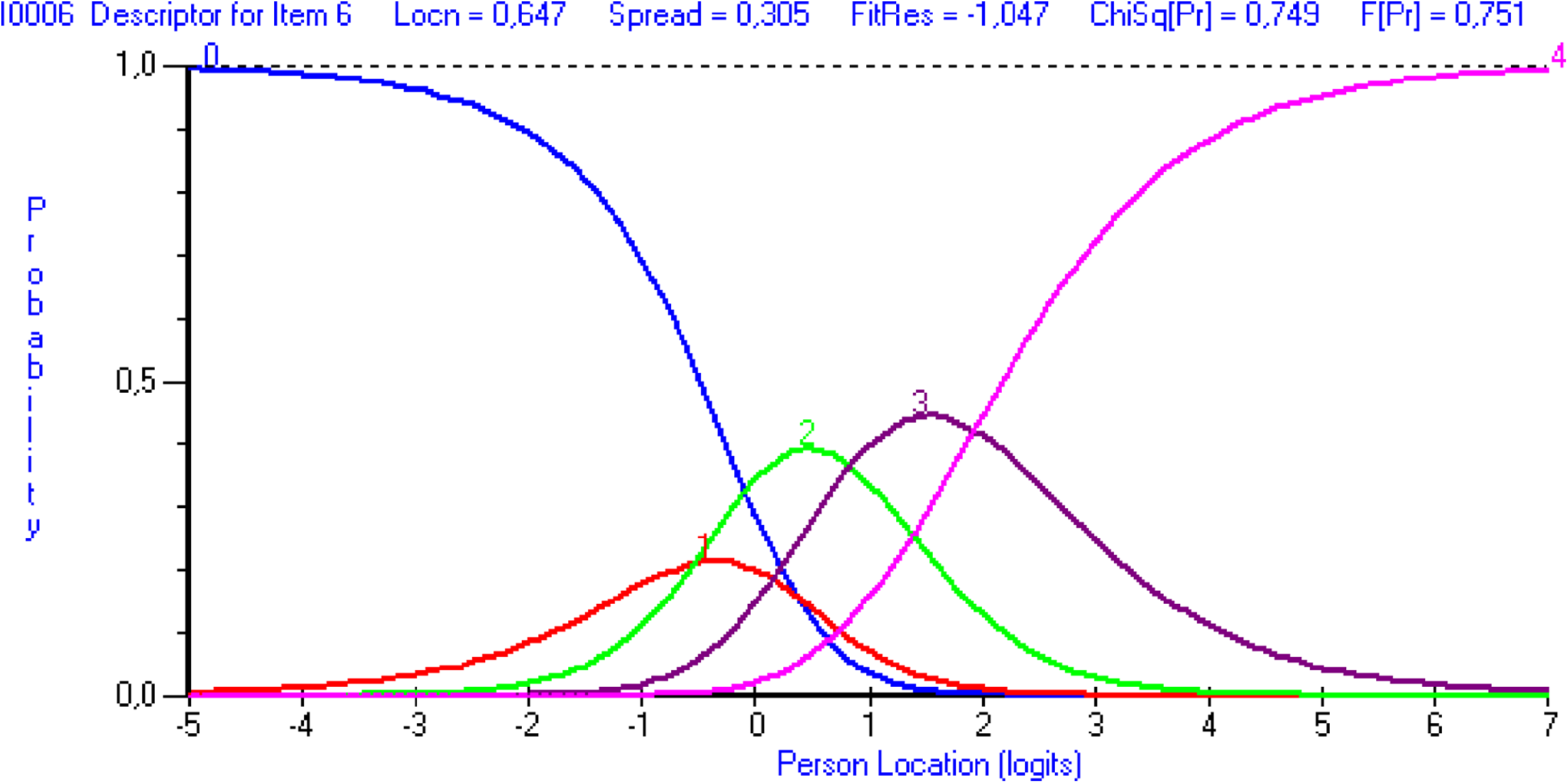

Supplement: Supplementary file 1 — Authors’ original file for figure 1 [file 12891_2014_2298_MOESM1_ESM.tif]

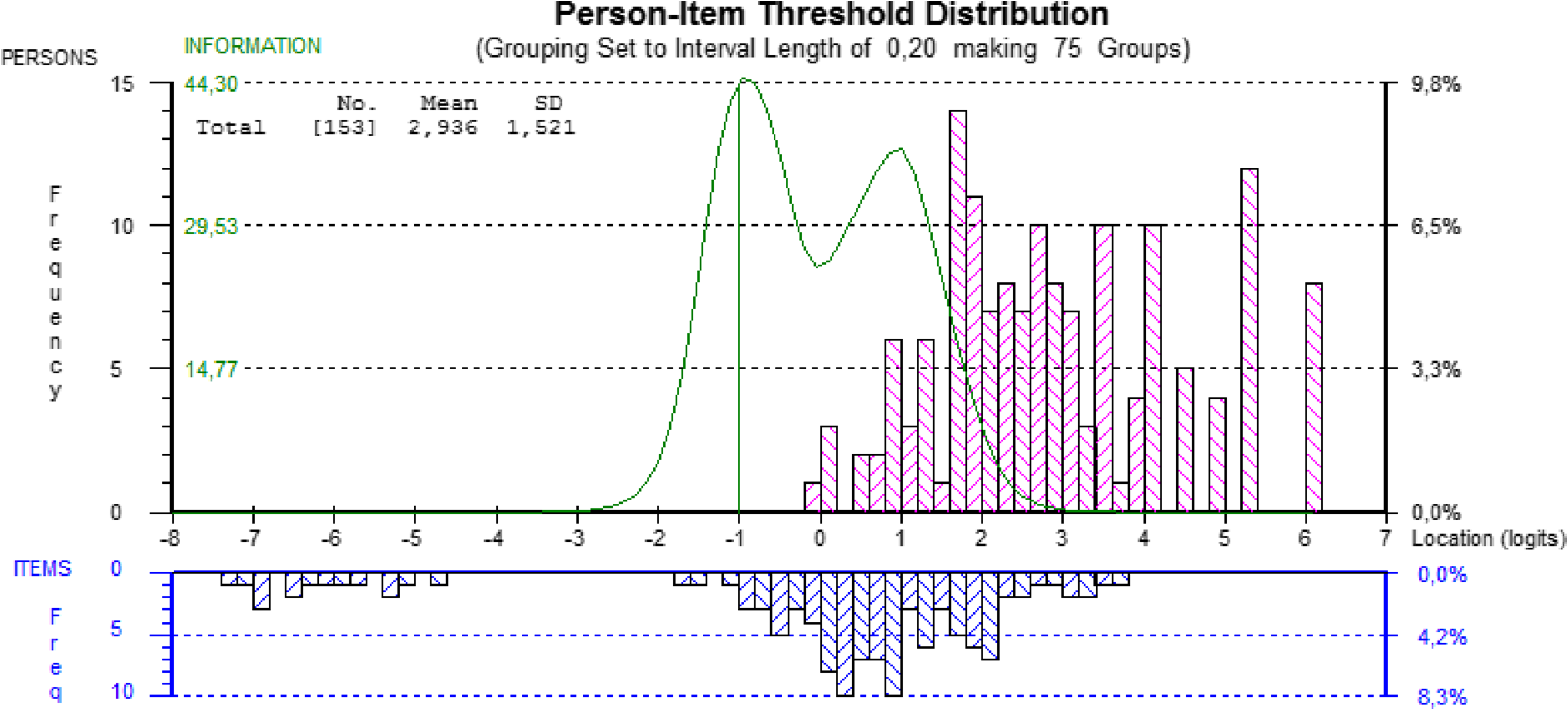

Supplement: Supplementary file 2 — Authors’ original file for figure 2 [file 12891_2014_2298_MOESM2_ESM.tif]

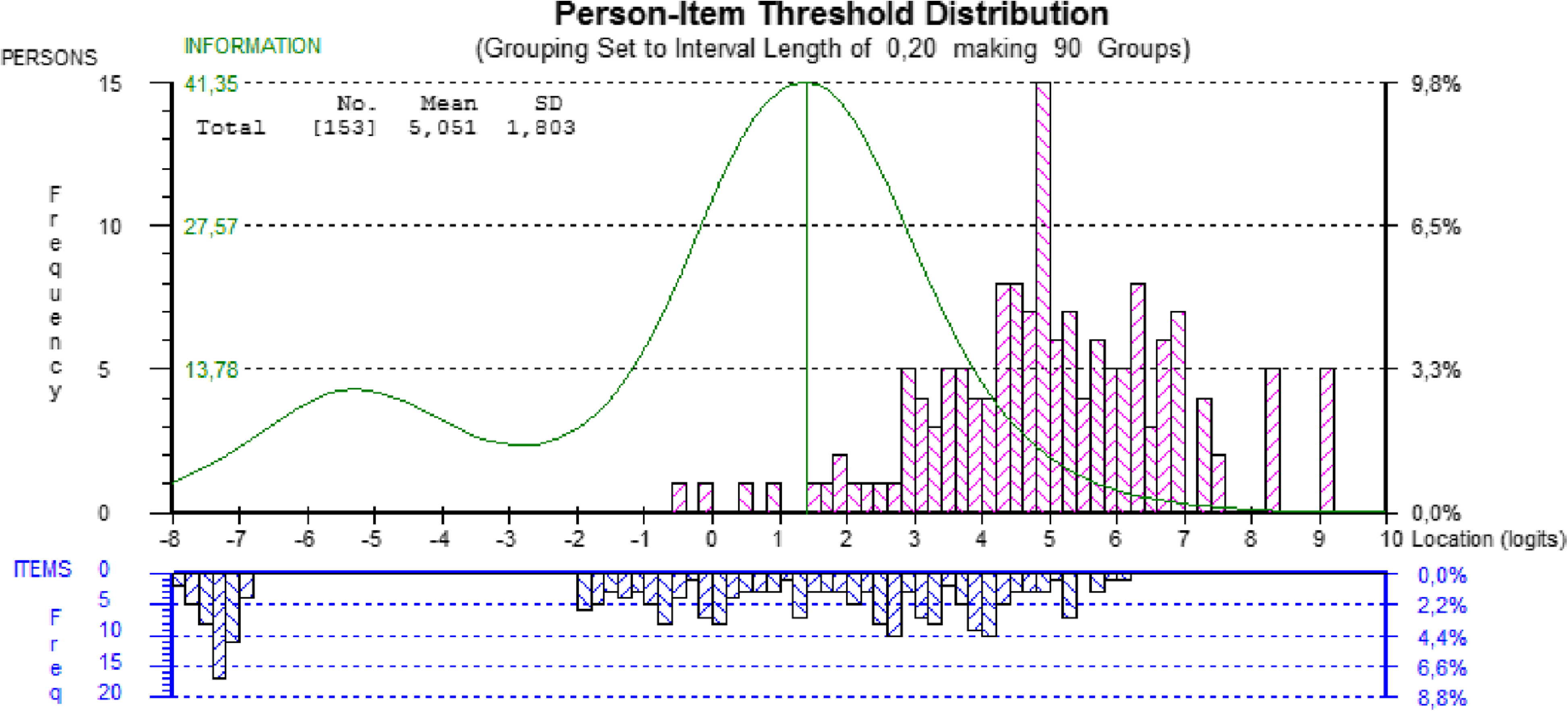

Supplement: Supplementary file 3 — Authors’ original file for figure 3 [file 12891_2014_2298_MOESM3_ESM.tif]
