# Supplementary material for: Psychometric evaluation of the Disabilities of the Arm, Shoulder and Hand (DASH) with Dupuytren’s contracture: validity evidence using Rasch modeling
Source: BMC Musculoskelet Disord. 2014 Oct 30;15:361. doi: 10.1186/1471-2474-15-361 (PMC4228176; doi:10.1186/1471-2474-15-361)
Supplement: Supplementary file 4 — Authors’ original file for figure 4 [file 12891_2014_2298_MOESM4_ESM.pdf]

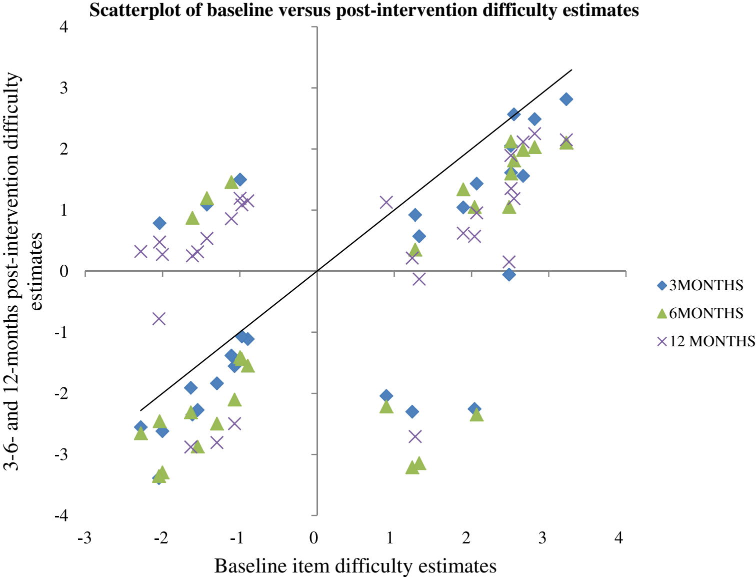

Scatterplot of baseline versus post-intervention difficulty estimates
4
3
2
1
0
-1
-2
-3
-4
-3
-2
-1
0
1
2
3
4
3-6- and 12-months post-intervention difficulty estimates
Baseline item difficulty estimates
3MONTHS
6MONTHS
12 MONTHS
